# Childhood trauma as a transdiagnostic factor in Borderline Personality Disorder and Eating Disorders: Relation with Impulsive-Unstable Symptomatology

**DOI:** 10.1192/j.eurpsy.2025.1915

**Published:** 2025-08-26

**Authors:** B. Marcos-Diaz, A. Gálvez-Marlín, N. Fuentes-García, P. Mola-Cardenes, J. M. López-Villatoro, A. B. Calvo-Calvo, M. Díaz-Marsá, J. L. Carrasco

**Affiliations:** 1 Hospital Clínico San Carlos; 2 Universidad Complutense de Madrid; 3 Centro de Investigación Biomédica en Red de Salud Mental, Madrid, Spain

## Abstract

**Introduction:**

Recent literature shows that childhood trauma might be associated with developing an eating disorder (ED) or borderline personality disorder (BPD) on the long term. These two disorders have a great comorbidity, and they share many common symptoms.

**Objectives:**

The aim of this study is to test if childhood trauma could be a transdiagnostic factor for both diagnostics, as well as analysing if trauma could be related to the severity of impulsive and instability symptomatology which characterize both of these diagnoses.

**Methods:**

The sample consisted of 45 patients with a diagnosis of either ED (n=21) or BPD (n=24). Childhood trauma was assessed using the CTQ (Childhood Trauma Questionnaire). Impulsive-unstable symptomatology was assessed using the BIS (Barrat’s Impulsivity Scale), CSV (Feeling of Emptiness Questionnaire) and STAXI (State-Trait Anger Expression Inventory), HARS (Hamilton’s Anxiety Rating Scale), MADRS (Montgomery-Asberg Depression Rating Scale). Differences between groups were measured for the CTQ using the *t* test. The relationship between test results and trauma was measured via regression analyses.

**Results:**

Both groups had high scores of emotional and sexual abuse, and the BPD group also showed high scores in emotional negligence. There were no statistically significant differences between groups relating to trauma symptoms (see *Figure 1*) Moreover, significant relationships were found between childhood trauma and higher levels of impulsivity (R^2^adj = 0.14; *p* = .006), feelings of emptiness (R^2^adj = 0.15; *p* = .005), anxiety (R^2^adj = 0.13; *p* = .008) and depression (R^2^adj = 0.08; *p* = .037).

**Image 1:**

**Figure:**
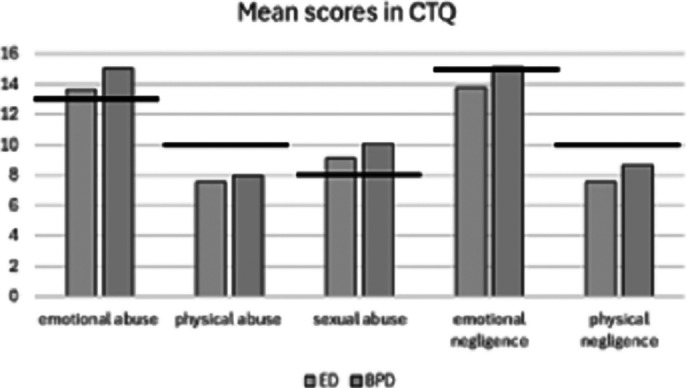

**Conclusions:**

Out preliminary study shows that childhood trauma is a transdiagnostic factor between BPD and EDs, and it’s related to the aggravation of impulsivity and instability symptomatology.

**Disclosure of Interest:**

None Declared

